# Bifunctional Nd-Doped LGSB Crystals: A Roadmap for Crystal Growth and Improved Laser Emission Performance in the NIR and Green Domains

**DOI:** 10.3390/ma18050964

**Published:** 2025-02-21

**Authors:** Alin Broasca, Madalin Greculeasa, Flavius Voicu, Cristina Gheorghe, Stefania Hau, Catalina Alice Susala, Lucian Gheorghe

**Affiliations:** National Institute for Laser, Plasma and Radiation Physics, 077125 Magurele, Romania; alin.broasca@inflpr.ro (A.B.);

**Keywords:** NIR laser emission, Nd, SFD, borate, bifunctional crystal, Czochralski

## Abstract

Herein we present a roadmap for tailoring the crystal growth conditions, near-infrared (NIR) laser emission, and self-frequency doubling (SFD) performances of newly developed Nd-doped La_x_Gd_y_Sc_4−x−y_(BO_3_)_4_ (Nd:LGSB) crystals. Three different Nd^3+^ doping concentrations of 2.3 at.%, 3.5 at.%, and 4.6 at.% were investigated. Considering their incongruent melting, special conditions were employed for the growth using the Czochralski technique. Laser emission performances at 1062 nm in the CW regime were evaluated for uncoated crystal samples with different orientations (**a**-cut, **c**-cut, and SFD-cut). The highest slope efficiency η_sa_ = 0.68 was obtained for the 4.6 at.% **c**-cut Nd:LGSB crystal, with a randomly polarized emission. The **a**-cut 4.6 at.% Nd:LGSB crystal delivered a linearly polarized beam with a slope efficiency η_sa_ = 0.63. The SFD-cut 2.3 at.% and 3.5 at.% Nd:LGSB crystals achieved slightly lower efficiencies of ~ 0.56. The SFD capabilities of 2.3 at.% and 3.5 at.% Nd:LGSB crystals were also explored. Green laser emission at ~531 nm was achieved with a diode-to-green conversion efficiency increasing significantly from 0.17% to 1.44%, respectively. These results demonstrate that the Nd-doping concentration, crystal orientation, and sample length of Nd:LGSB crystals, must be carefully selected depending on the specific requirements of the intended application.

## 1. Introduction

Since the introduction of the first ruby laser crystal in 1960, there has been continuous development in laser technology. Understanding the importance of the laser emission characteristics and identifying suitable laser active media are essential for advancing laser applications. Currently, lasers find extensive applications across diverse fields such as information technology and communications [1,2,3], surgery and medicine [4,5,6,7], military uses [8,9], materials processing technologies [10,11,12], laser ignition [13,14,15], scientific research, and more. To meet the criteria imposed by these applications, the laser emission must cover a wide spectral range from ultraviolet (UV) to infrared (IR), including the visible range (VIS). Typically, semiconductor-based laser diodes (LDs) serve as the most prevalent pumping sources for solid-state lasers. However, LDs do not cover certain spectral regions within the visible range, such as the 535–620 nm range, known as the “green gap”. Furthermore, the inferior spectral quality of laser diode emissions, characterized by wider bandwidth and beam divergence, dictates their utility primarily as pumping sources for solid-state lasers. In contrast to laser diodes, solid-state lasers offer the capability to alter the emission wavelength by changing the activator ion and demonstrate superior laser beam quality.

In the realm of solid-state lasers, extensive research has been conducted on the development of new laser crystals. To advance the development and testing of new laser crystals, it is imperative to first find the optimal methods for their growth and fabrication. Enhancing the crystal growth conditions requires a comprehensive understanding of both the physical properties of the molten and solid states. Moreover, enhancing crystal quality relies on elucidating the formation mechanism of growth defects, thereby enabling the implementation of effective strategies to eliminate them. Concurrently, the demand for novel crystals in advanced technologies has stimulated enhancements in crystal growth techniques, although these are primarily focusing on a few pivotal crystals. The Czochralski (CZ) growth technique stands out as the predominant method for growing laser crystals, owing to its versatility across a wide spectrum of materials and melting points. This technique spans from mixed compounds like nitrates to single oxides such as sapphire and yttria, encompassing diverse materials like germinates, fluorides, molybdates, tantalates, and garnets. To address the challenges encountered during crystal growth, the standard CZ growth process has been modified numerous times to improve the quality of the grown crystals. To date, sapphire, yttrium aluminum garnet (YAG), and yttrium or gadolinium vanadates (YVO_4_ and GdVO_4_, respectively) crystals are widely recognized as primary hosts, owing to their unique chemical and physical properties, including suitable crystal fields for active ions and good thermal properties.

In the domain of high-efficiency diode-pumped solid-state (DPSS) lasers, Nd and Yb ions are the primary candidates [16,17], both having a typical laser emission wavelength in the range of ~1 μm. Neodymium trivalent ions (Nd^3+^) have garnered attention due to their four-level emission scheme, which effectively avoids signal reabsorption from the fundamental level. This characteristic renders Nd^3+^ more advantageous for achieving amplified gain, thus enhancing its suitability for various laser applications. Among the Nd-doped single crystals, Nd:YAG, Nd:YVO_4_, and Nd:GdVO_4_ have gained relevance. Typically, Nd:YVO_4_ and Nd:YAG continuous-wave (CW) lasers longitudinally pumped with laser diodes around 0.81 μm exhibit slope efficiencies of approximately 60% concerning the absorbed pump power (P_abs_), by using anti-reflection (AR) coated crystals, thus yielding optimal results. Enhanced slope efficiencies, up to 80% in Nd:YVO_4_, were achieved using alternative pumping geometries or resonant pumping as close as possible to the emitting laser level [18,19].

To further push the boundaries of solid-state laser technology and address the need for versatile and efficient laser sources at a wider wavelength range, researchers have focused on exploring bifunctional, laser, and nonlinear optical (NLO) crystals. These crystals possess the remarkable ability to not only generate laser emissions but also to self-frequency double the lasing wavelength, effectively extending the spectral coverage into previously inaccessible regions, including the VIS. In general, a bifunctional crystal must satisfy multiple criteria, including high quantum efficiency, good thermal conductivity, efficient absorption at the pump wavelength, efficient laser emission (e.g., in the near-infrared (NIR) range to obtain self-frequency-doubling (SFD) laser emission in the VIS domain), and phase-matching properties with minimal losses. Additionally, the crystal should be capable of attaining high optical quality at substantial dimensions. However, incorporating significant quantities of trivalent rare-earth ions in bifunctional crystals can impose challenges to crystal growth, leading to degradation of optical quality. One of the most promising bifunctional crystals is the Nd^3+^-doped yttrium aluminum borate (Nd:YAB) crystal, which, for a doping concentration with Nd^3+^ ions of 4 at.%, has demonstrated a green output power of 225 mW at a pump power of 1.6 W at 807 nm from a laser diode [20]. Nevertheless, due to its incongruent melting nature, Nd:YAB crystals can only be grown via the flux method, making it exceedingly challenging to obtain sufficiently large and high-quality single crystals. To address these growth limitations, research has shifted towards scandium derivatives with a huntite-type structure, characterized by the general formula LnSc_3_(BO_3_)_4_ (Ln = lanthanide), which have the potential to circumvent the growth issues encountered with Nd:YAB crystals. From this class of crystals, the newly developed Nd-doped La_x_Gd_y_Sc_4−x−y_(BO_3_)_4_ (Nd:LGSB) crystal stands out, which offers the possibility to be grown with high quality and large dimensions by the Czochralski method, and also fulfills all the necessary criteria to be an effective bifunctional crystal.

This review explores the latest advancements in the development of Nd:LGSB-type crystals with tailored properties for efficient laser emission in the NIR range and self-frequency doubling (SFD) in the VIS domain.

## 2. Crystal Growth, Compositional, and Structural Analyses

Considering that La_x_Gd_y_Sc_4−x−y_(BO_3_)_4_ (LGSB) crystals have incongruent melting [21], the starting melt compositions as well as the experimental conditions for the growth of Nd:LGSB-type crystals by the CZ technique must be carefully selected. Given that the ionic radius of Nd^3+^ ions in a six-fold coordination environment is closer to that of La^3+^ ions rather than Gd^3+^ ions [22], the starting composition of the pure LGSB crystal was adjusted to incorporate the Nd doping ions with respect to La ions, while maintaining the Gd content constantly. Nd:LGSB crystals doped with different concentrations of Nd^3+^ ions of 2.5 at.%, 3.8 at.%, and 5 at.% in the starting melt have been grown by the CZ technique, employing specific growth conditions tailored to optimize the quality of the grown crystal. Due to the peritectic nature of Nd:LGSB crystals, the starting melt compositions were selected to facilitate the direct crystallization of the trigonal phase (space group R*32*). Therefore, the selected starting melt compositions were La_0.653_Nd_0.025_Gd_0.572_Sc_2.75_(BO_3_)_4_ (2.5 at.% Nd:LGSB), La_0.640_Nd_0.038_Gd_0.572_Sc_2.75_(BO_3_)_4_ (3.8 at.% Nd:LGSB), and La_0.628_Nd_0.05_Gd_0.572_Sc_2.75_(BO_3_)_4_ (5.0 at.% Nd:LGSB). La_2_O_3_, Nd_2_O_3_, Gd_2_O_3_, Sc_2_O_3_ (of 99.999% purity), and B_2_O_3_ (of 99.98% purity) oxide powders were used as raw materials. To compensate for the B_2_O_3_ evaporation during the sintering of starting compounds and the crystal growth processes, an additional 5 wt.% of B_2_O_3_ was added to the stoichiometric amounts.

**Figure 1:**
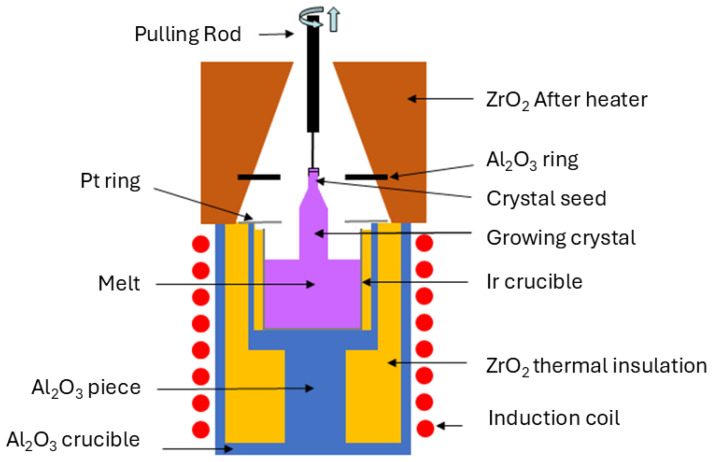
Schematic representation of the thermal setup used for the growth of Nd:LGSB crystals by the Czochralski method.

The growth experiments were performed in a CZ furnace equipped with inductive radio-frequency (RF) heating, which must maintain stable and precise temperature control throughout the crystal growth process to ensure uniform crystal quality. An iridium crucible measuring 30 mm in height and diameter was utilized, and therefore, all crystal growth processes were conducted in a static N_2_ atmosphere to prevent oxidation of the crucible. A difficult challenge is given by the high viscosity of the melt, along with its tendency for phase separation and vitrification. This can be surpassed by an intense stirring of the melt to maintain its uniformity, typically achieved by inducing high radial thermal gradients within the melt. However, this approach unavoidably leads to overheating and increased B_2_O_3_ vapor concentration. The condensation of B_2_O_3_ vapors onto the growing crystal surface presents a significant risk, as liquid B_2_O_3_ droplets formed through condensation can infiltrate the high-temperature zone, dissolving the crystal and disrupting the crystallization process, potentially causing failure and detachment of the crystal from the melt. To address this challenge, a special thermal setup (Figure 1) was engineered to adjust the radial and vertical thermal gradients to optimal values. The thermal setup managed to provide sufficiently large thermal gradients to stabilize the growth interface while keeping the evaporations at a low level, thus avoiding the constitutional supercooling. In this aim, the main particularity of the thermal setup consists of adding one Pt ring and one Al_2_O_3_ ring situated at distances of 2 mm and 20 mm above the top of the crucible, respectively [21].

**Figure 2:**
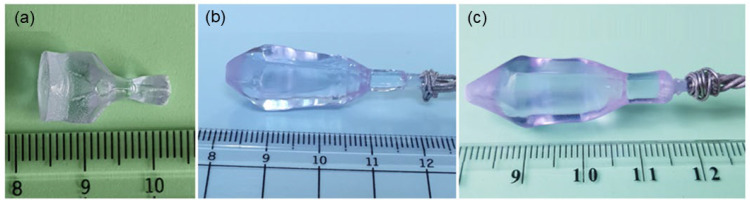
Nd:LGSB crystals grown from the starting melt compositions (**a**) La_0.653_Nd_0.025_Gd_0.572_Sc_2.75_(BO_3_)_4_, (**b**) La_0.640_Nd_0.038_Gd_0.572_Sc_2.75_(BO_3_)_4_, and (**c**) La_0.628_Nd_0.05_Gd_0.572_Sc_2.75_(BO_3_)_4_ [23,24].

Under these conditions, three incongruent melting Nd:LGSB single crystals were successfully grown from the starting melt compositions La_0.653_Nd_0.025_Gd_0.572_Sc_2.75_(BO_3_)_4_, La_0.640_Nd_0.038_Gd_0.572_Sc_2.75_(BO_3_)_4_, and La_0.628_Nd_0.05_Gd_0.572_Sc_2.75_(BO_3_)_4_ using the Czochralski crystal growth method. Nd:LGSB crystal seeds oriented along the *c*-axis were used for the growth of all the crystals and the pulling and rotation rates were optimized at 2 mm/h and 8–10 rpm, respectively. The growth temperatures were measured to be ~1490 ± 10 °C. As can be observed in Figure 2 [23,24], the as-grown crystals have excellent quality, characterized by a high transparency and the absence of visible defects. Their distinctive light purple color is associated with the incorporation of Nd^3+^ ions in the crystal structure. All the grown crystals present a hexagonal transversal section with clearly $\left\{2\;\bar{1}\;\bar{1\;}0\right\}$ and $\left\{1\;1\;\bar{2\;}0\right\}$ facets, characteristic of ***c***-axis grown huntite-type crystals.

The chemical composition and uniformity along the ***c***-axis of the as-grown crystals were examined using the inductively coupled plasma atomic emission spectroscopy (ICP-AES) method, employing samples obtained from various areas of the crystals, including the shoulder, body, and tail of the obtained crystals. The boron and oxygen contents were considered to be in stoichiometric quantities, consistent with huntite-type crystals. The ICP-AES measurements revealed that all crystals have good homogeneity along the growth direction. Table 1 presents the compositions of the Nd:LGSB grown crystals. From these results, the effective segregation coefficient of Nd^3+^ ions in the LGSB crystals was determined to be k_eff_ = 0.92. Consequently, the concentrations of Nd^3+^ ions effectively incorporated into the grown crystals were found to be 2.3 at.%, 3.5 at.%, and 4.6 at.%, respectively.

The room temperature X-ray powder diffraction (XRPD) spectra of the La_0.745_Nd_0.023_Gd_0.452_Sc_2.78_(BO_3_)_4_ (2.3 at.% Nd:LGSB), La_0.733_Nd_0.035_Gd_0.452_Sc_2.78_(BO_3_)_4_ (3.5 at.% Nd:LGSB), and La_0.721_Nd_0.046_Gd_0.452_Sc_2.781_(BO_3_)_4_ (4.6 at.% Nd:LGSB) crystals revealed the existence of a single trigonal phase (space group *R32*) for all the crystals. The lattice constants were found to be very close to each other, being almost similar to those of the undoped LGSB crystal, having the values of *a* = 9.793 Å and *c* = 7.954 Å [21,23,24]. According to reference [24], La^3+^, Nd^3+^ and a significant fraction of Gd^3+^ ions occupy trigonal prismatic sites connected to each other by [BO_3_]^3−^ triangles, while Sc^3+^ and a small fraction of Gd^3+^ ions occupy the octahedral sites. This structural arrangement ensures a large distance of ~6.2 Å between the closest Nd^3+^ ions, being advantageous for incorporating high doping concentration without quenching effects.

## 3. Nonlinear Optical Properties

**Figure 3:**
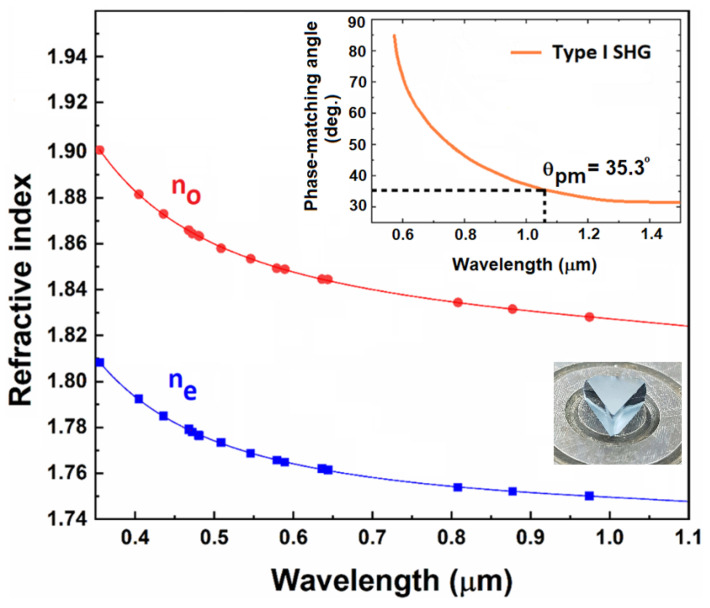
Dispersion of the refractive indices of the 3.5 at.% Nd:LGSB crystal. The experimental data points are shown as full dots, while the solid curves represent the Sellmeier fit of these data points. The insets show the phase matching curve for type-I SHG and the prism used to measure the refractive index [24].

In the field of NLO crystals, the ability to achieve phase matching for a specific wavelength is crucial. This ability allows NLO crystals to be effectively used in various technological applications. Nd:LGSB-type crystals are negative uniaxial crystals (the ordinary refractive index, n_o_, is higher than the extraordinary refractive index, n_e_) having the optical axis parallel to the crystallographic ***c***-axis. The wavelength dispersion of refractive indices between 350 nm and 975 nm at room temperature was determined by the minimum deviation method. The measured values of the n_o_ and n_e_ were fitted by the following Sellmeier equation:

$$n_{i}^{2}\left(\lambda \right)=A+\frac{B}{\lambda^{2}-C}-D\lambda^{2},$$

where *i* denotes the ordinary (o) or extraordinary (e) indices, *λ* is the wavelength expressed in µm and A, B, C, and D are the Sellmeier coefficients. As anticipated, the refractive indices were found to be similar for all the Nd:LGSB investigated crystals, being close to those of the undoped LGSB crystal [21]. Figure 3 shows the dispersion of the refractive indices along with the Sellmeier fit and the phase-matching curve for type I SHG in the 3.5 at.% Nd:LGSB crystal. As can be observed in Figure 3 [24], the phase-matching angle for type-I SHG of 1064 nm radiation was found to be θ = 35.3°. As expected, this angle closely matches the reported value for the pure LGSB crystal (θ = 35.8°, [21]). Furthermore, the minimum fundamental wavelength capable of generating type-I SHG in the Nd:LGSB-type crystals is 570 nm, which is higher than that reported for the YAB crystal (490.5 nm, [25]).

Using the refractive index values, phase-matching angles (θ, φ), walk-off angle (ρ), and angular (Δθ × L) and spectral (Δλ × L) acceptances were determined for the SHG of the 1064 nm fundamental wavelength in type-I phase-matching configuration [25]. Additionally, the d_11_ nonlinear coefficient was calculated following the method described in references [26,27]. The results obtained are similar for all the Nd:LGSB crystals and are presented in Table 2. It can be observed that the values obtained for the Czochralski-grown Nd:LGSB (4.6 at.%) crystal are very similar to those of pure LGSB and almost as good as those of YAB crystal grown by the top-seeded solution growth (TSSG) method [28]. These findings demonstrate that the incorporation of Nd^3+^ ions has a negligible effect on the NLO properties of the LGSB host crystal. This outcome aligns with expectations, as it is well-established that the NLO properties of crystals are predominantly governed by the spatial arrangement of their anionic groups [29]. In the case of Nd:LGSB crystals, the NLO properties specifically originate from the [BO_3_] planar triangular anionic groups.

## 4. Optical Properties and Laser Emission Performances

### 4.1. Spectroscopic Investigation

Optical spectroscopy is an indispensable tool in studying and evaluating the interaction of an optically active crystal with electromagnetic radiation, allowing the evaluation of its prospects as a laser crystal. Therefore, 2.3 at.% Nd:LGSB, 3.5 at.% Nd:LGSB, and 4.6 at.% Nd:LGSB crystals were investigated by optical transmission measurements at room temperature (300 K), absorption and emission measurements at 300 K and low temperature (10 K), and emission kinetic measurements at 300 K.

The polarized absorption and emission spectra of Nd^3+^ ions in Nd:LGSB crystals were recorded at low (10 K) and room temperature (300 K) using an experimental setup that included Jarrell Ash Czerny-Turner and Horiba Jobin-Yvone monochromators equipped with S20, S1 photomultipliers, and a Ge photodiode, along with a lock-in amplifier connected to a computer for data acquisition. For room temperature measurements, the emission spectra were obtained under excitation at ~800 nm using a Xe lamp. The emission cross-sections for both σ and π polarizations were determined using the Fuchtbauer–Ladenburg method [32]. For the low-temperature measurements, the samples were mounted on a cold stand and cooled with a cycle helium refrigerator (ARS-2HW). The fluorescence lifetime of ^4^F_3/2_ (Nd^3+^) level was measured at room temperature under excitation at 808 nm using an optical parametric oscillator (OPO) laser (OPOTEK RADIANT 355 LD). The decay signals were displayed and analyzed using a Tektronix 2024B oscilloscope

#### 4.1.1. Optical Transmission

**Figure 4:**
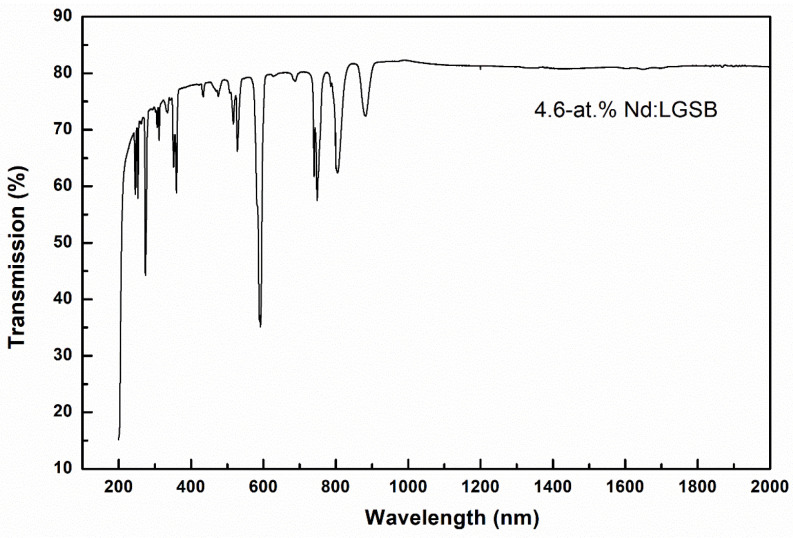
Optical transmission spectrum of the 4.6 at.% Nd:LGSB crystal [23].

The optical transmission measurements of the Nd:LGSB crystals at 300 K were performed using a Varian Cary UV-vis-NIR spectrophotometer on ***c***-cut crystal samples (radiation propagation through the crystal sample along the ***c***-axis) with a thickness of about 1 mm cut from the grown crystals and polished to laser quality. The obtained spectra showed that all grown crystals are characterized by a broad optical transparency range, between 200 nm and above 2.0 μm, having a transmittance of over 82% in the 1 μm wavelength range, thus being very promising for obtaining efficient laser emission at 1.06 μm of Nd^3+^-doped ions. To exemplify, the optical transmission spectrum obtained on the 4.6 at.% Nd:LGSB crystal sample is presented in Figure 4 [23]. The absorption lines in the wavelength range from 244 nm to 312 nm are characteristics of Gd^3+^ ions, while the lines in the range from 330 nm to 900 nm are typical to *f-f* transitions of Nd^3+^ ions [33], thus proving the incorporation of Nd^3+^ doping ions into the LGSB host crystal.

#### 4.1.2. Room Temperature Absorption and Emission Spectra

**Figure 5:**
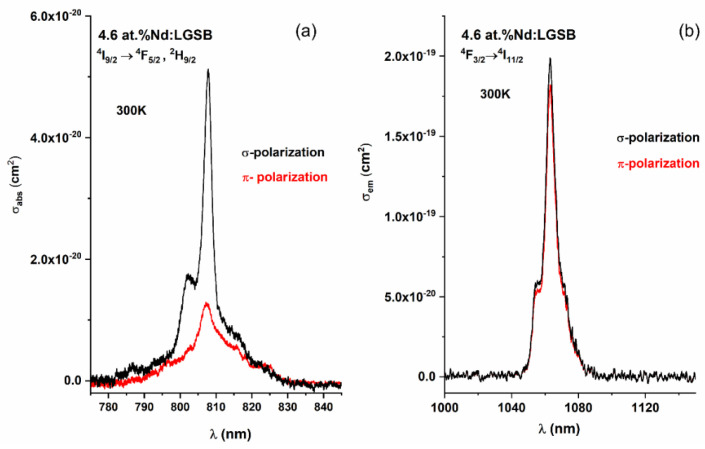
Polarized absorption (**a**) and emission (**b**) cross-sections of 4.6 at.% Nd:LGSB crystal [35].

Useful information regarding the pump absorption efficiency of Nd-based laser crystals can be achieved by investigating the absorption cross-sections (*σ_abs_*) in the 800 nm range, corresponding to the strongly absorbing ^4^I_9/2_→ ^4^F_5/2_, ^2^H_9/2_ transition. Thus, the obtained values of *σ*_abs_ for the 3.5 at.%, and 4.6 at.% Nd:LGSB crystals are presented in Table 3. As observed, the maximum values were obtained for the 4.6 at.% Nd:LGSB crystal, due to the higher doping concentration with Nd^3+^ ions. For a more expressive visualization, the polarized absorption cross-section spectra of the 4.6 at.% Nd:LGSB crystal are shown in Figure 5a. In the case of σ polarization, the peak absorption occurs at 807.8 nm, with a value of 5.1 × 10^−20^ cm^2^ and a full-width at half-maximum (FWHM) of 3.2 nm. In contrast, π polarization exhibits a maximum absorption at 807.1 nm with a reduced cross-section of 1.3 × 10^−20^ cm^2^, approximately four times smaller than for σ polarization, but with a significantly broader FWHM of 8.3 nm. The emission cross-sections (*σ*_em_) corresponding to ^4^F_3/2_ → ^4^I_11/2_ transition, for both *σ* and *π* polarizations, are also presented in Table 3. The emission cross-sections at 1063 nm (shown in Figure 5b) are very close for both 4.6 at.% and 3.5 at.% Nd:LGSB crystals, the maximum value being 2.1 × 10^−19^ cm^2^ in *σ* polarization in the case of 4.6 at.% Nd:LGSB crystal, while in *π* polarization both crystals had a value of 1.8 × 10^−19^ cm^2^ [23,24]. The values of σ_em_ are higher than those of Nd:YAB (1.11 × 10^−19^ cm^2^ in σ polarization) [25] and Nd:LSB (1.3 × 10^−19^ cm^2^ in σ polarization) [34], proving the high potential of the Nd:LGSB crystals to generate efficient laser emission at 1063 nm. The emission bandwidths (FWHM) of the 4.6 at.% Nd:LGSB crystal were determined to be 6.5 nm and 7.3 nm for *σ* and *π* polarization, respectively. These values are significantly larger than those of Nd:YAG (~0.5 nm), highlighting the potential of Nd:LGSB for generating ultrashort laser pulses ranging from a few picoseconds to hundreds of femtoseconds.

**Table 3:** Absorption and emission cross-sections of Nd:LGSB crystals compared with Nd:LSB [34], Nd:YAB [25], and Nd:YAG [36] crystals.

Crystal	σ_abs_ (cm^2^)		σ_em_ (cm^2^)	
	λ = 808 nm		λ = 1063 nm	
	σ	π	σ	π
3.5% Nd:LGSB [24]	4.7 × 10^−20^	1.1 × 10^−20^	1.9 × 10^−19^	1.8 × 10^−19^
4.6% Nd:LGSB [35]	5.1 × 10^−20^ FWHM = 3.2 nm	1.3 × 10^−20^ FWHM = 8.3 nm	2.1 × 10^−19^ FWHM = 6.5 nm	1.8 × 10^−19^ FWHM = 7.3 nm
Nd:LSB [34]	7.1 × 10^−20^ FWHM = 3 nm	1.9 × 10^−20^ FWHM = 5 nm	1.3 × 10^−19^ FWHM = 4 nm	5 × 10^−20^ FWHM = 4 nm
Nd:YAB [25]	2.58 × 10^−20^	0.94 × 10^−20^	1.11 × 10^−19^	1.18 × 10^−19^
Nd:YAG [36]	7 × 10^−20^ FWHM = 0.8 nm		3.3 × 10^−19^ FWHM = 0.5 nm	

#### 4.1.3. Low-Temperature Absorption and Emission Spectra

**Figure 6:**
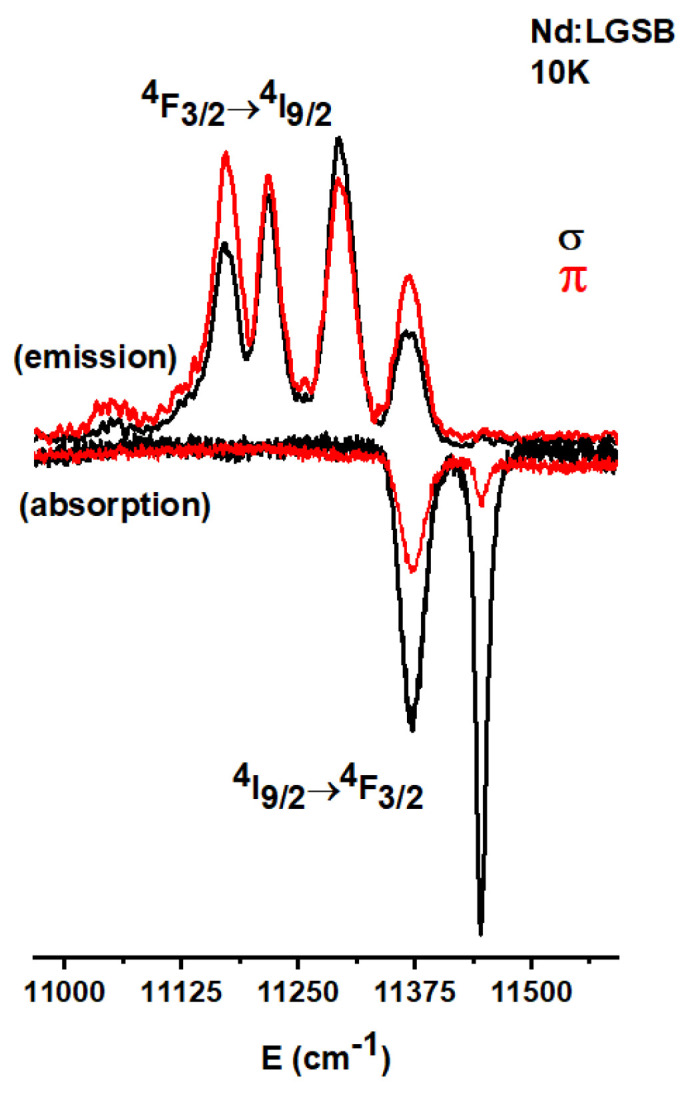
Low-temperature polarized absorption and emission spectra of Nd:LGSB crystals [23].

The absorption and emission spectra of Nd^3+^ ions at 10 K were recorded to provide detailed insights into the energy level structure of Nd^3+^ ions within the LGSB matrix. As expected, the spectra were found to be similar for all the crystals. In particular, the absorption spectra of the 4.6 at.% Nd:LGSB crystal sample, measured in the 880 nm spectral region, are shown in Figure 6 [23]. The spectra display two distinct absorption lines (11,372 and 11,446 cm^−1^) corresponding to the Stark levels of the ^4^F_3/2_ manifold. The low-temperature emission spectra in the 870–900 nm range reveal five Stark levels corresponding to the ^4^I_9/2_ manifold (0, 78, 154, 198, and 328 cm^−1^), suggesting that, at 10 K, only the first Stark level of the ^4^F_3/2_ manifold is populated. These observations indicate the presence of a single type of Nd^3+^ centers, implying that Nd^3+^ ions exclusively substitute La^3+^ ions and not Sc^3+^ ions in the crystal lattice, which can be deduced considering that the ionic radius of Nd^3+^ (r_Nd_ = 0.983 Å) is closer to that of La^3+^ (r_La_ = 1.032 Å) than to that of Sc^3+^ (r_Sc_ = 0.745 Å) in six-fold coordination.

#### 4.1.4. Fluorescence Lifetime and Quantum Efficiency

Considering that a higher concentration of Nd^3+^ doping ions influences the fluorescence lifetime, the 4.6 at.% Nd crystal was selected for these investigations to evaluate the performances of Nd:LGSB crystals under conditions where concentration effects are most significant. Thus, the fluorescence decay of the ^4^F_3/2_ metastable level was measured for the 4.6 at.% Nd:LGSB crystal at room temperature under 808 nm excitation using an OPO laser. The experimental fluorescence lifetime (τ_exp_) was determined to be 144 µs. This value is consistent with similar measurements for the Nd:LSB crystal (τ_exp_ = 150 µs [34]). The radiative lifetime (τ_rad_) of the 4.6 at.% Nd:LGSB crystal was calculated from polarized absorption spectra [32] and was found to be 243 µs. Therefore, the fluorescence quantum efficiency (η = τ_exp_/τ_rad_) was determined to be 59.2%, being comparable to the Nd:LSB crystal (η = 60.2%) [34].

### 4.2. Laser Emission Performances

Laser emission performances in the NIR domain at 1.06 μm of the Nd:LGSB crystals have been studied under various operating regimes, including free-running, Q-switching, and mode-locking [23,24,35,37]. Herein, for a relevant comparison, we focus on laser emission under a continuous-wave (CW) regime. Laser experiments were performed on oriented samples cut from the grown crystals and without any antireflective coatings, as follows: oriented along the **c**-axis direction (**c**-cut), along the **a**-axis direction (**a**-cut), and along the phase-matching direction for type I SHG of 1.06 μm radiation (SFD-cut). The pumping was performed using a fiber-coupled laser diode with emission centered at 807 nm, operated in the CW regime. The pump beam was focused on the samples using a pair of achromatic doublets, each of them with a focal length of 40 mm. The crystal samples were wrapped in indium foil and mounted in copper holders to ensure proper thermal contact. Cooling was provided by a recirculation system integrated with a Peltier element for optimum temperature control. A compact linear plane-plane resonator with a length of 10 mm was employed. The high reflectivity mirror (HRM), through which optical pumping was performed, featured an antireflective coating (reflectivity, R > 0.998) at the fundamental emission wavelength λ_em_ or its second harmonic (λ_2ω_ = 0.53 μm) and high transmittance (transmission, T > 0.98) at the pump wavelength λ_p_. Output coupling mirrors (OCMs) with different transmittances (T_OC_ = 0.01, 0.024, 0.03, 0.05, 0.10, and 0.15) were used to obtain high-efficiency laser emission. Two dichroic mirrors were placed after the optical resonator to select only the laser emission in NIR (at 1.06 μm) and that in the VIS spectrum (at 0.53 μm) in the case of SFD experiments. A Spiricon camera (model SP620U, spectral range 190–1100 nm) was used to record the intensity distribution of the laser beam. The polarization of the laser beam was determined using a Glan–Taylor polarizer (extinction ratio greater than 100,000:1). For each Nd-doping concentration and sample orientation, the laser emission was investigated under similar experimental conditions. Therefore, the analysis aims to provide a comprehensive comparison of laser performances across the doping concentrations and sample orientation.

#### 4.2.1. NIR Laser Emission

**Figure 7:**
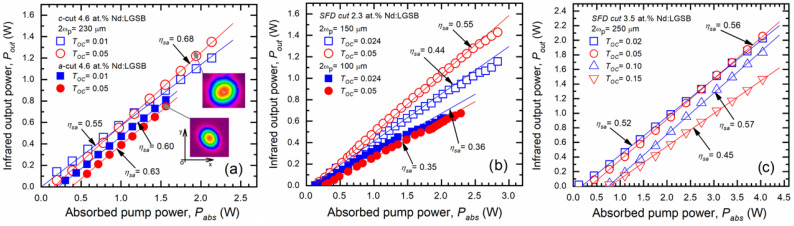
CW laser operation at 1062 nm of 4.6 at.% Nd:LGSB (**a**), 2.3 at.% Nd:LGSB (**b**), and 3.5 at.% Nd:LGSB (**c**) crystals. The insets of figure (**a**) show the near field distributions for the indicated points [24,35,37].

In the case of 4.6 at.% Nd:LGSB crystal, two samples with ***c***-cut and ***a***-cut orientations were investigated. For the ***c***-cut 4.6 at.% Nd:LGSB crystal, a maximum output power (P_out_) of 1.35 W at 1062 nm was achieved using an OCM with a transmission of 0.05, at an absorbed pump power (P_abs_) of 2.14 W. This corresponds to an optical-to-optical efficiency (η_oa_) of 0.63 and a slope efficiency (η_sa_) of 0.68 (Figure 7a). The polarization analysis revealed that the laser beam emitted by the ***c***-cut sample was randomly polarized. The near-field distribution showed a circular beam profile with diameters (Ox × Oy axes) of 4.8 mm × 4.9 mm (1/e^2^ definition). For the ***a***-cut 4.6 at.% Nd:LGSB crystal, the maximum P_out_ was 0.81 W, obtained with an OCM of T_OC_ = 0.03, for P_abs_ = 1.55 W, leading to an optical-to-optical efficiency of η_oa_ = 0.52 and a corresponding slope efficiency of η_sa_ = 0.60. When the OCM with T_OC_ = 0.05 was used, the slope efficiency improved to η_sa_ = 0.63, but the output power decreased to P_out_ = 0.75 W [35]. The laser beam from the ***a***-cut crystal was linearly polarized with a polarization ratio of 100:1, emitting a σ-polarized beam, which corresponds to the polarization with the highest emission cross-section. The near-field distribution exhibited a slightly elliptical shape, with beam diameters of 2.2 mm × 2.3 mm, likely caused by temperature gradients or differing thermal expansion coefficients along the crystal axes. A comparison between the results obtained for the ***c***-cut and ***a***-cut 4.6 at.% Nd:LGSB samples reveals distinct differences in performance and beam characteristics:

(i) Output Power and Efficiency:
  - The **c**-cut sample achieved a higher maximum output power (P_out_) of 1.35 W compared to 0.81 W for the **a**-cut sample. This represents a significant improvement in output power for the **c**-cut orientation.
  - η_oa_ was also higher for the **c**-cut sample (0.63) than for the **a**-cut sample (0.52). Similarly, η_sa_ for the **c**-cut sample of 0.68 exceeded that of the **a**-cut sample of 0.63 corresponding to the OCM with T_OC_ = 0.05.
(ii) Polarization:
  - The **c**-cut sample emitted a randomly polarized laser beam, while the **a**-cut sample produced a linearly polarized beam with a polarization ratio of 100:1. The **a**-cut crystal emitted σ-polarized light, which is advantageous due to the higher emission cross-section for this polarization.
(iii) Beam Profile:
  - The near-field distribution of the **c**-cut sample exhibited a circular beam shape with diameter of 4.8 mm × 4.9 mm (1/e^2^ definition). In contrast, the **a**-cut sample displayed a slightly elliptical beam profile with diameters of 2.2 mm × 2.3 mm. This ellipticity may result from temperature gradients or differences in thermal expansion coefficients along the axes of the uniaxial crystal.

Therefore, the **c**-cut 4.6 at.% Nd:LGSB sample demonstrated superior performance in terms of output power and efficiency, making it more suitable for applications requiring high-power, randomly polarized laser beams. However, the **a**-cut crystal offers the advantage of linearly polarized emission, which is critical for applications requiring well-defined polarization states. The choice between **c**-cut and **a**-cut orientations should therefore depend on the specific requirements of the intended application, balancing power output and polarization control.

In the case of 2.3 at.% and 3.5 at.% Nd:LGSB crystals [24,37], the NIR laser emission properties were investigated using SFD-cut (θ = 35.3°, φ = 60°) samples. The 2.3 at.% Nd:LGSB sample was investigated for two different beam diameters of the pumping radiation (Figure 7b). Under pumping conditions with a tightly focused beam (2ω_p_ = 100 μm) and an OCM transmission of T_OC_ = 0.05, the crystal emitted a maximum output power of P_out_ = 0.67 W at an absorbed pump power of P_abs_ = 2.3 W, corresponding to an optical-to-optical efficiency of η_oa_ = 0.29. The slope efficiency achieved in this configuration was η_sa_ = 0.35. The pump absorption efficiency of the 2.3 at.% Nd:LGSB crystal under these conditions was η_abs_ = 0.64. With an OCM transmission of T_OC_ = 0.024, the slope efficiency increased to η_sa_ = 0.35. However, a saturation of the emission was observed for P_abs_ values exceeding ~2.0 W, most likely because of thermal effects within the 2.3 at.% Nd:LGSB crystal. Laser performance was further enhanced by employing a pump beam with a larger diameter of 2ω_p_ = 150 μm. In this configuration, using an OCM with T_OC_ = 0.024, the output power increased to P_out_ = 1.15 W at P_abs_ = 2.83 W, with a slope efficiency of η_sa_ = 0.44. When an OCM with T_OC_ = 0.05 was used, the output power increased to P_out_ = 1.43 W, corresponding to an optical efficiency of η_oa_ = 0.50 and an improved slope efficiency of η_sa_ = 0.55.

For the SFD-cut 3.5 at.% Nd:LGSB sample, a maximum P_out_ of 2.02 W was achieved using an OCM with T_OC_ = 0.02 for an P_abs_ of 4.04 W. This corresponds to an optical-to-optical efficiency η_oa_ = 0.50 and a slope efficiency of η_sa_ = 0.52 (Figure 7c). When the OCM transmission was increased to T_OC_ = 0.05, the maximum output power improved to P_out_ = 2.1 W for the same absorbed pump power, resulting in an optical-to-optical efficiency of η_oa_ = 0.52 and a higher slope efficiency of η_sa_ = 0.56. Further increasing the OCM transmission to T_OC_ = 0.1 slightly improved the slope efficiency to η_sa_ = 0.57. Even with an OCM transmission of T_OC_ = 0.15, the crystal delivered watt-level output power, achieving a P_out_ = 1.47 W. The laser threshold for this sample was measured to be 360 mW, and a Findlay-Clay analysis indicated a resonator round-trip loss (L_i_) of approximately 1%. Compared to the 2.3 at.%, the 3.5 at.% Nd:LGSB sample exhibited superior performance, in terms of delivered output power at 1.06 μm.

A comparison of the laser emission performance of the well-known Nd-based laser crystals and the newly developed Nd:LGSB crystals is presented in Table 4.

The main results and conclusions are presented below:

- 2 at.% Nd:YAG achieved a slope efficiency of η_sa_ = 0.56 and a maximum output power of P_out_ = 12.3 W. While Nd:YAG excels in output power, the *c*-cut 4.6 at.% Nd:LGSB outperformed it in slope efficiency (η_sa_ = 0.68), making Nd:LGSB more attractive for energy-efficient applications.
- 0.7 at.% Nd:YVO_4_ reached a high slope efficiency of η_sa_ = 0.66, comparable to the *c*-cut 4.6 at.% Nd:LGSB (η_sa_ = 0.68), but with higher output power. However, Nd:YVO_4_ lacks the bifunctional capabilities of Nd:LGSB.
- 6 at.% Nd:YAB achieved a η_sa_ = 0.38 being significantly lower than the η_sa_ = 0.56 obtained by the 3.5 at.% Nd:LGSB with the same crystallographic orientation. This demonstrates the superior lasing performance of Nd:LGSB crystals.
- 10 at.% Nd:LSB crystal delivered a maximum P_out_ = 0.32 W with η_sa_ = 0.55, being outperformed by the Nd:LGSB in both slope efficiency and output power.

Thus, the Nd:LGSB crystals exhibit excellent NIR laser performances, with high slope efficiencies and competitive output powers.

#### 4.2.2. SFD Laser Emission

The green laser emission performances of Nd-doped crystals by SFD processes of fundamental NIR emission are strongly influenced by the partial reabsorption of the green-generated radiation by the Nd ions. In SFD lasers, the Nd^3+^ concentration in the host crystal and the crystal length are two important factors that determine the SFD output power and optical efficiency at a fixed pump beam waist. Generally, for a given Nd^3+^ concentration, there is an optimum crystal length which normally decreases with an increasing Nd^3+^ concentration. Therefore, an equilibrium between the Nd ions concentration and the length of the crystal sample must be made to minimize green reabsorption and optimize SFD efficiency. Another important limiting factor for the SFD performance of a crystal is constituted by the thermal effects resulting from the pump-induced thermal loading, which affects both IR performance and infrared-to-visible conversion efficiency. The thermal load of the SFD crystal leads to a change in its refractive indices that induces a phase mismatch between fundamental and second harmonic waves, thus resulting in the decrease in the infrared-to-visible conversion efficiency. In this regard, the pump beam diameter plays an important role in the management of the crystal’s thermal loading. Considering that a Nd-ion concentration of 4.6 at.% is too high for obtaining green laser emission by SFD in Nd-doped huntite-type crystals (e.g., Nd:YAB crystal [42]), the laser emission properties of both 2.3 at.% and 3.5 at.% Nd:LGSB crystals were investigated in SFD configuration to generate green emission at 531 nm in the CW regime. The experiments carried out aimed to determine the optimum conditions for SFD and compare the efficiency, output power, and overall performance of the Nd:LGSB crystals with the two doping concentrations, highlighting the intrinsic potential of Nd:LGSB crystals for green laser emission.

**Figure 8:**
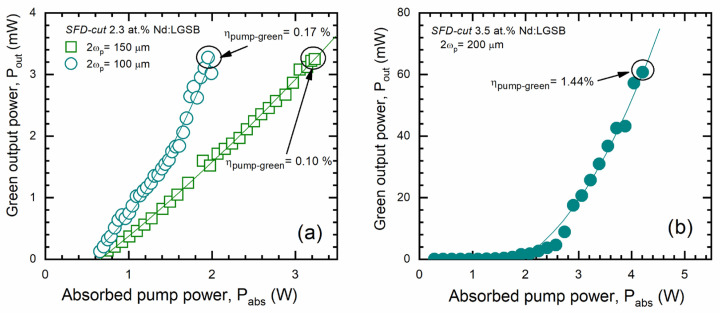
Green output power at 0.53 μm generated by the 2.3 at.% Nd:LGSB (**a**) and 3.5 at.% Nd:LGSB (**b**) crystals [24,37].

Similarly to the case of the NIR laser experiments, the 2.3 at.% Nd:LGSB SFD-cut crystal sample with a length of 3.7 mm was investigated for two different beam diameters of the pumping radiation to determine optimum pumping conditions [37]. For the pump beam with a larger diameter of 2ω_p_ = 150 μm, the 2.3 at.% Nd:LGSB sample generated green laser emission with an output power of P_out_ = 3.3 mW for an absorbed pump power of P_abs_ = 3.2 W, corresponding to a diode-to-green conversion efficiency of 0.10% (Figure 8a). In contrast, with a tightly focused pump beam (2ω_p_ = 100 μm), the same green power (P_out_ = 3.3 mW) was obtained at a lower absorbed pump power of P_abs_ = 1.95 W. In this configuration, the diode-to-green conversion efficiency increased to 0.17%. However, further increasing the pump power in this configuration resulted in roll-over behavior, followed by a rapid decline in green emission. This phenomenon can be attributed to thermal effects induced in the 2.3 at.% Nd:LGSB crystal due to the tighter focusing of the pump beam.

In the case of the 3.5 at.% Nd:LGSB crystal [24], a maximum green SFD power of P_out_ = 60 mW for an absorbed pump power of P_abs_ = 4.20 W, corresponding to a diode-to-green conversion efficiency of 1.44%, was obtained for a sample having a length of 6 mm using an optimal diameter of the pump beam of 2ω_p_ = 200 μm, as shown in Figure 8b. Compared to the 2.3 at.% Nd:LGSB crystal, the efficiency increased remarkably from 0.17% to 1.44% (of about 8.5 times), thus underlining the important role of optimizing the Nd doping concentration and crystal length in enhancing the SFD performances. Even though the results obtained in terms of diode-to-green conversion efficiency are currently modest compared to the Nd:YAB crystal (pump-to-visible conversion efficiency of 14%) [20], they are very encouraging considering that new bifunctional Nd:LGSB-type crystals are in an early stage of research. A priori, the appropriate concentration of Nd ions should be between the two tested concentrations, probably closer to 3.5 at.%.

## 5. Conclusions

Bifunctional Nd:LGSB crystals with incongruent melting and different concentrations of Nd^3+^ dopant ions were grown by the Czochralski method. The as-grown crystals have excellent quality, characterized by high transparency and the absence of visible defects. All the grown crystals present a hexagonal transversal section with clearly $\left\{2\;\bar{1}\;\bar{1\;}0\right\}$ and $\left\{1\;1\;\bar{2\;}0\right\}$ facets, characteristic of ***c***-axis grown huntite-type crystals. The chemical composition and uniformity along the ***c***-axis revealed that all crystals have a good homogeneity along the growth direction, and the concentrations of Nd^3+^ ions effectively incorporated into the grown crystals were found to be 2.3 at.%, 3.5 at.%, and 4.6 at.%, respectively. The refractive indices were found to be similar for all the Nd:LGSB investigated crystals, being close to those of the undoped LGSB crystal. Thus, the incorporation of Nd^3+^ ions has a negligible effect on the NLO properties of the LGSB host crystal. The phase-matching angle for type-I SHG of 1064 nm fundamental radiation was found to be θ = 35.3°. The optical transmission measurements showed that all grown crystals are characterized by a broad optical transparency range, between 200 nm and above 2.0 μm, having a transmittance of over 82% in the 1 μm wavelength range, thus being very promising for obtaining efficient laser emission at ~1.06 μm. The spectroscopic investigations of the Nd:LGSB crystals revealed that Nd^3+^ ions substitute only La^3+^ cations in the LGSB crystal matrix, as well as their high potential to generate efficient laser emission in the NIR domain at ~1.06 μm. The laser emission performances in the CW regime at 1062 nm were evaluated for each Nd-doping concentration using uncoated crystal samples with different crystallographic orientations, such as ***a***-cut, ***c***-cut, and *SFD*-cut. In terms of slope efficiency, the *c*-cut 4.6 at.% Nd:LGSB has permitted us to obtain the highest value of η_sa_ = 0.68, while the optical-to-optical efficiency reached η_oa_ = 0.63 at high absorbed pump power, P_abs_ = 2.14 W. The NIR emission of the ***c***-cut Nd:LGSB crystal was randomly polarized, while the ***a***-cut 4.6 at% Nd:LGSB crystal delivered a linearly polarized output beam with highest laser slope efficiency of η_sa_ = 0.63. In the case of *SFD*-cut 3.5 at% Nd:LGSB crystal sample, slightly lower slope and optical-to-optical efficiencies (η_sa_ = 0.56 and η_oa_ = 0.52) were reached, and increased output powers of ~2 W were obtained. These results demonstrate that the Nd-doping concentration and crystal orientation must be selected depending on the specific requirements of the intended application, balancing slope efficiency, power output, and also the control of the polarization state. Moreover, the bifunctionality of Nd:LGSB for self-frequency doubling further differentiates it from traditional laser crystals like Nd:YAG and Nd:YVO_4_, making it a promising candidate for green laser generation and NIR applications. The laser emission performances at ~531 nm in SFD configuration under CW regime of both 2.3 at.% and 3.5 at.% Nd:LGSB crystals were also investigated. The diode-to-green conversion efficiency improved significantly from 0.17% in the case of 2.3 at.% Nd:LGSB crystal to 1.44% (of about 8.5 times increase) in the case of 3.5 at.% Nd:LGSB crystal, highlighting the importance of optimizing Nd doping concentration and crystal length for enhanced SFD performance. While diode-to-green conversion efficiencies remain modest, these results are promising given the early research stage of bifunctional Nd:LGSB-type crystals. To conclude, this work represents a roadmap for selecting the appropriate Nd-doping concentration, crystallographic orientation, and sample length of the Czochralski-grown Nd:LGSB crystals, for NIR laser and/or green SFD specific applications.

## Figures and Tables

**Table 1:** Compositions of the Nd:LGSB crystals measured by ICP-AES [23,24].

Starting Melt Composition	Crystal Composition
La_0.653_Nd_0.025_Gd_0.572_Sc_2.75_(BO_3_)_4_	La_0.745_Nd_0.023_Gd_0.452_Sc_2.78_(BO_3_)
La_0.640_Nd_0.038_Gd_0.572_Sc_2.75_(BO_3_)_4_	La_0.733_Nd_0.035_Gd_0.452_Sc_2.78_(BO_3_)_4_
La_0.628_Nd_0.05_Gd_0.572_Sc_2.75_(BO_3_)_4_	La_0.721_Nd_0.046_Gd_0.452_Sc_2.781_(BO_3_)_4_

**Table 2:** Comparison of the NLO properties for type-I SHG of 1064 nm fundamental radiation for Czochralski-grown LGSB, Nd:LGSB, and flux-grown YAB crystals [23].

Crystal	(θ, φ) (deg.)	Δn	ρ (deg.)	Δθ × L (deg. × cm)	Δλ × L (nm × cm)	d_11_ (pm/V)
LGSB [21]	(35.8, 60)	0.078	2.60	0.030	0.79	1.35
Nd:LGSB (4.6 at.%)	(35.3, 60)	0.077	2.43	0.033	0.71	1.35
YAB [30]	(30.8, 60)	0.071	2.23	0.035	1.43	1.69 [31]

**Table 4:** Laser emission performances of different Nd-doped crystals under CW diode-pumping [24,35,37].

Crystal	Concentration (at.%)	Orientation	Length (mm)	η_sa_	P_out max_ (W)
Nd:LGSB [this work]	4.6	***a***-cut	3	0.60	0.81
		***c***-cut	6.1	0.68	1.35
	2.3	SFD-cut	3.7	0.55	0.67
	3.5	SFD-cut	6	0.56	2.1
Nd:YVO_4_ [38]	3.15	***a***-cut	1	0.35	0.11
Nd:YVO_4_ [39]	0.7	-	4	0.66	12.1
Nd:YAG [39]	2	[111]	6	0.56	12.3
Nd:LSB [40]	10	-	1	0.55	0.32
Nd:YAB [41]	6	SFD-cut	4	0.38	3.2

## Data Availability

The data presented in this study are available upon request from the corresponding author. The data are not publicly available due to technical limitations.
